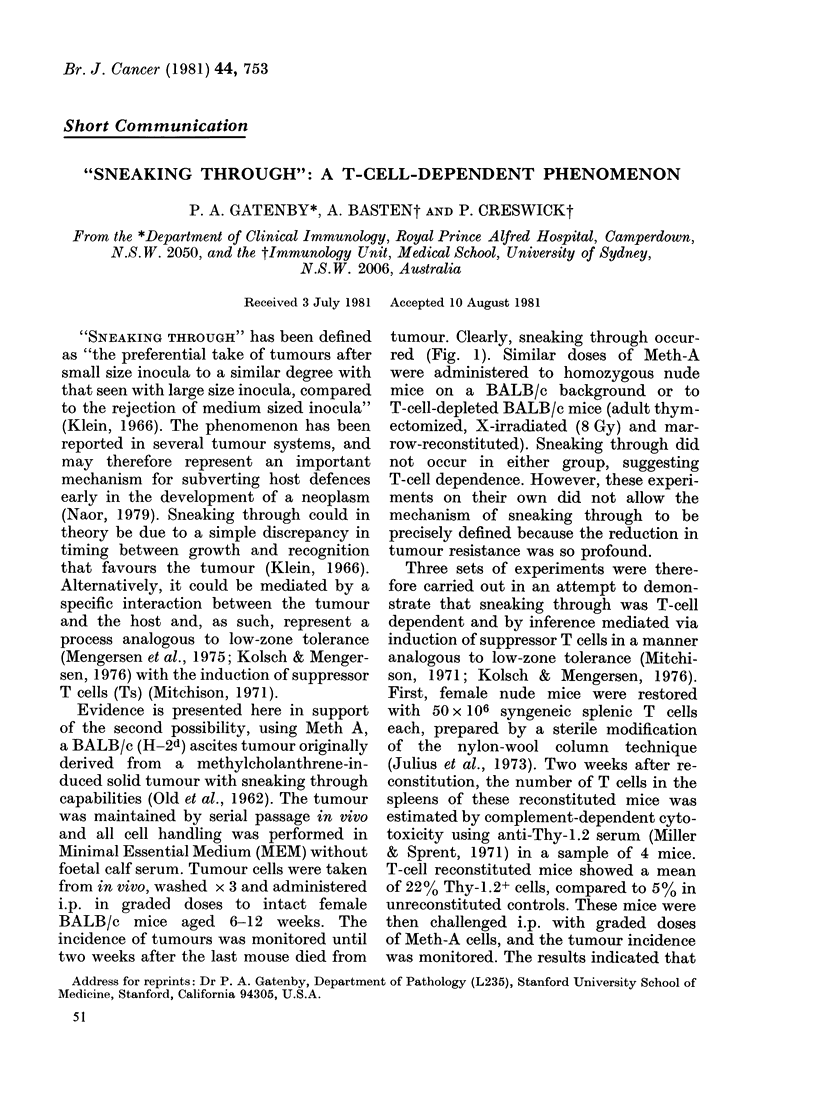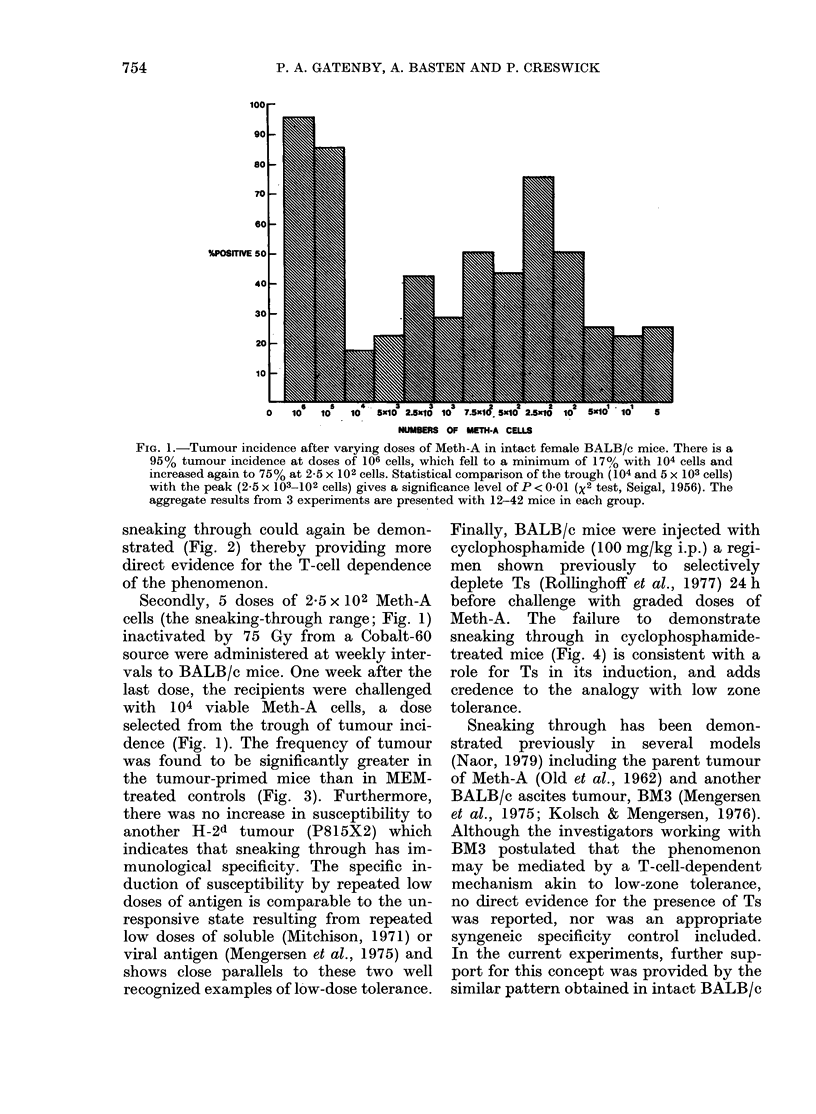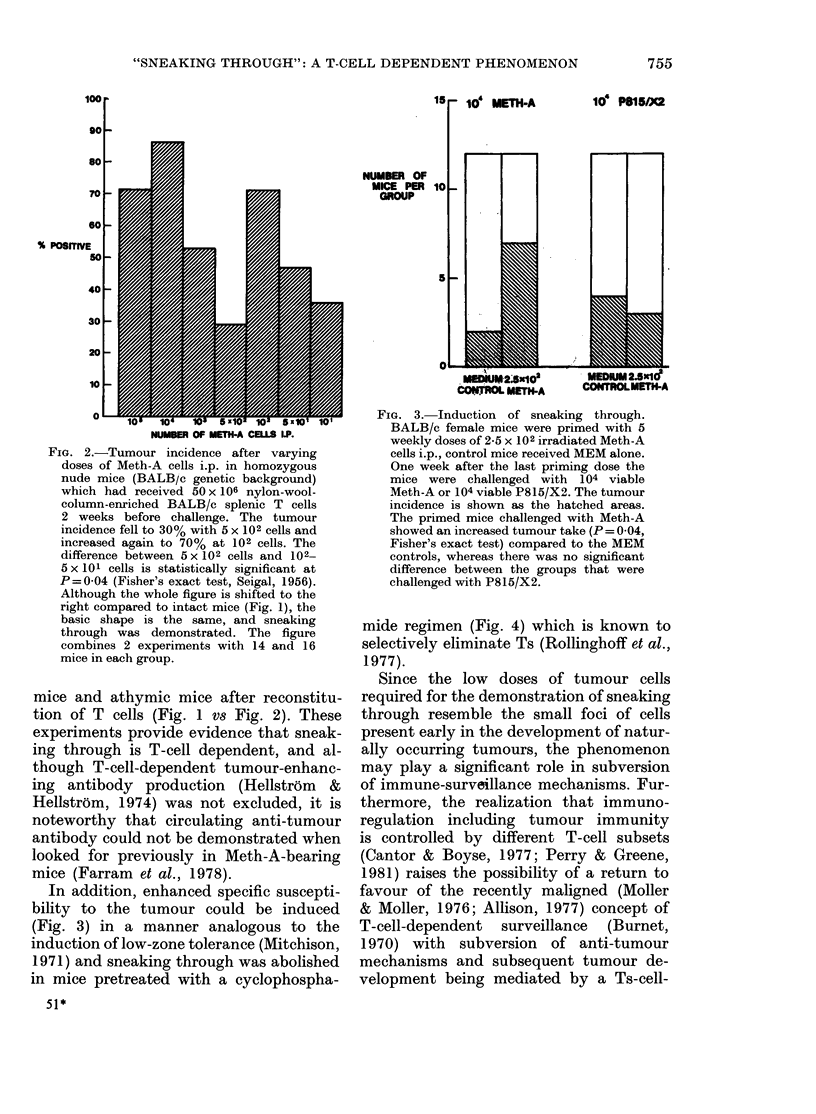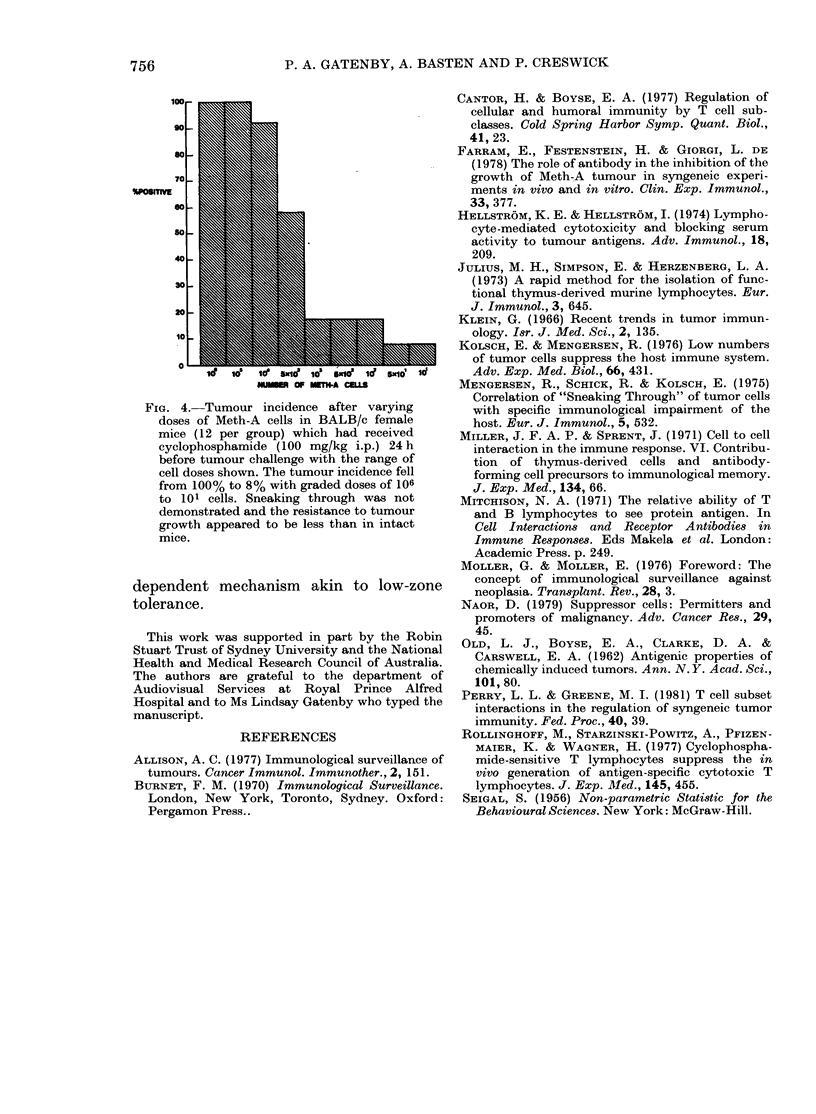# "Sneaking through": a T-cell-dependent phenomenon.

**DOI:** 10.1038/bjc.1981.264

**Published:** 1981-11

**Authors:** P. A. Gatenby, A. Basten, P. Creswick


Br. J. Cancer (1981) 44, 753

### Short Communication

### "SNEAKING THROUGH": A T-CELL-DEPENDENT PHENOMENON

P. A. GATENBY*, A. BASTEN† AND P. CRESWICK†

*From the *Department of Clinical Immunology, Royal Prince Alfred Hospital, Camperdown, N.S.W. 2050, and the †Immunology Unit, Medical School, University of Sydney, N.S.W. 2006, Australia*

Received 3 July 1981 Accepted 10 August 1981

"Sneaking through" has been defined as "the preferential take of tumours after small size inocula to a similar degree with that seen with large size inocula, compared to the rejection of medium sized inocula" (Klein, 1966). The phenomenon has been reported in several tumour systems, and may therefore represent an important mechanism for subverting host defences early in the development of a neoplasm (Naor, 1979). Sneaking through could in theory be due to a simple discrepancy in timing between growth and recognition that favours the tumour (Klein, 1966). Alternatively, it could be mediated by a specific interaction between the tumour and the host and, as such, represent a process analogous to low-zone tolerance (Mengersen *et al.*, 1975; Kolsch & Mengersen, 1976) with the induction of suppressor T cells (Ts) (Mitchison, 1971).

Evidence is presented here in support of the second possibility, using Meth A, a BALB/c (H–$2^d$) ascites tumour originally derived from a methylcholanthrene-induced solid tumour with sneaking through capabilities (Old *et al.*, 1962). The tumour was maintained by serial passage *in vivo* and all cell handling was performed in Minimal Essential Medium (MEM) without foetal calf serum. Tumour cells were taken from *in vivo*, washed × 3 and administered i.p. in graded doses to intact female BALB/c mice aged 6–12 weeks. The incidence of tumours was monitored until two weeks after the last mouse died from tumour. Clearly, sneaking through occurred (Fig. 1). Similar doses of Meth-A were administered to homozygous nude mice on a BALB/c background or to T-cell-depleted BALB/c mice (adult thymectomized, X-irradiated (8 Gy) and marrow-reconstituted). Sneaking through did not occur in either group, suggesting T-cell dependence. However, these experiments on their own did not allow the mechanism of sneaking through to be precisely defined because the reduction in tumour resistance was so profound.

Three sets of experiments were therefore carried out in an attempt to demonstrate that sneaking through was T-cell dependent and by inference mediated via induction of suppressor T cells in a manner analogous to low-zone tolerance (Mitchison, 1971; Kolsch & Mengersen, 1976). First, female nude mice were restored with $50 \times 10^6$ syngeneic splenic T cells each, prepared by a sterile modification of the nylon-wool column technique (Julius *et al.*, 1973). Two weeks after reconstitution, the number of T cells in the spleens of these reconstituted mice was estimated by complement-dependent cytotoxicity using anti-Thy-1.2 serum (Miller & Sprent, 1971) in a sample of 4 mice. T-cell reconstituted mice showed a mean of 22% Thy-$1.2^+$ cells, compared to 5% in unreconstituted controls. These mice were then challenged i.p. with graded doses of Meth-A cells, and the tumour incidence was monitored. The results indicated that

Address for reprints: Dr P. A. Gatenby, Department of Pathology (L235), Stanford University School of Medicine, Stanford, California 94305, U.S.A.

FIG. 1.—Tumour incidence after varying doses of Meth-A in intact female BALB/c mice. There is a 95% tumour incidence at doses of $10^6$ cells, which fell to a minimum of 17% with $10^4$ cells and increased again to 75% at $2{\cdot}5 \times 10^2$ cells. Statistical comparison of the trough ($10^4$ and $5 \times 10^3$ cells) with the peak ($2{\cdot}5 \times 10^3$–$10^2$ cells) gives a significance level of $P < 0{\cdot}01$ ($\chi^2$ test, Seigal, 1956). The aggregate results from 3 experiments are presented with 12–42 mice in each group.

sneaking through could again be demonstrated (Fig. 2) thereby providing more direct evidence for the T-cell dependence of the phenomenon.

Secondly, 5 doses of $2{\cdot}5 \times 10^2$ Meth-A cells (the sneaking-through range; Fig. 1) inactivated by 75 Gy from a Cobalt-60 source were administered at weekly intervals to BALB/c mice. One week after the last dose, the recipients were challenged with $10^4$ viable Meth-A cells, a dose selected from the trough of tumour incidence (Fig. 1). The frequency of tumour was found to be significantly greater in the tumour-primed mice than in MEM-treated controls (Fig. 3). Furthermore, there was no increase in susceptibility to another H-$2^d$ tumour (P815X2) which indicates that sneaking through has immunological specificity. The specific induction of susceptibility by repeated low doses of antigen is comparable to the unresponsive state resulting from repeated low doses of soluble (Mitchison, 1971) or viral antigen (Mengersen *et al.*, 1975) and shows close parallels to these two well recognized examples of low-dose tolerance.

Finally, BALB/c mice were injected with cyclophosphamide (100 mg/kg i.p.) a regimen shown previously to selectively deplete Ts (Rollinghoff *et al.*, 1977) 24 h before challenge with graded doses of Meth-A. The failure to demonstrate sneaking through in cyclophosphamide-treated mice (Fig. 4) is consistent with a role for Ts in its induction, and adds credence to the analogy with low zone tolerance.

Sneaking through has been demonstrated previously in several models (Naor, 1979) including the parent tumour of Meth-A (Old *et al.*, 1962) and another BALB/c ascites tumour, BM3 (Mengersen *et al.*, 1975; Kolsch & Mengersen, 1976). Although the investigators working with BM3 postulated that the phenomenon may be mediated by a T-cell-dependent mechanism akin to low-zone tolerance, no direct evidence for the presence of Ts was reported, nor was an appropriate syngeneic specificity control included. In the current experiments, further support for this concept was provided by the similar pattern obtained in intact BALB/c

FIG. 2.—Tumour incidence after varying doses of Meth-A cells i.p. in homozygous nude mice (BALB/c genetic background) which had received $50 \times 10^6$ nylon-wool-column-enriched BALB/c splenic T cells 2 weeks before challenge. The tumour incidence fell to 30% with $5 \times 10^2$ cells and increased again to 70% at $10^2$ cells. The difference between $5 \times 10^2$ cells and $10^2$–$5 \times 10^1$ cells is statistically significant at $P = 0{\cdot}04$ (Fisher's exact test, Seigal, 1956). Although the whole figure is shifted to the right compared to intact mice (Fig. 1), the basic shape is the same, and sneaking through was demonstrated. The figure combines 2 experiments with 14 and 16 mice in each group.

FIG. 3.—Induction of sneaking through. BALB/c female mice were primed with 5 weekly doses of $2{\cdot}5 \times 10^2$ irradiated Meth-A cells i.p., control mice received MEM alone. One week after the last priming dose the mice were challenged with $10^4$ viable Meth-A or $10^4$ viable P815/X2. The tumour incidence is shown as the hatched areas. The primed mice challenged with Meth-A showed an increased tumour take ($P = 0{\cdot}04$, Fisher's exact test) compared to the MEM controls, whereas there was no significant difference between the groups that were challenged with P815/X2.

mice and athymic mice after reconstitution of T cells (Fig. 1 *vs* Fig. 2). These experiments provide evidence that sneaking through is T-cell dependent, and although T-cell-dependent tumour-enhancing antibody production (Hellström & Hellström, 1974) was not excluded, it is noteworthy that circulating anti-tumour antibody could not be demonstrated when looked for previously in Meth-A-bearing mice (Farram *et al.*, 1978).

In addition, enhanced specific susceptibility to the tumour could be induced (Fig. 3) in a manner analogous to the induction of low-zone tolerance (Mitchison, 1971) and sneaking through was abolished in mice pretreated with a cyclophosphamide regimen (Fig. 4) which is known to selectively eliminate Ts (Rollinghoff *et al.*, 1977).

Since the low doses of tumour cells required for the demonstration of sneaking through resemble the small foci of cells present early in the development of naturally occurring tumours, the phenomenon may play a significant role in subversion of immune-surveillance mechanisms. Furthermore, the realization that immunoregulation including tumour immunity is controlled by different T-cell subsets (Cantor & Boyse, 1977; Perry & Greene, 1981) raises the possibility of a return to favour of the recently maligned (Moller & Moller, 1976; Allison, 1977) concept of T-cell-dependent surveillance (Burnet, 1970) with subversion of anti-tumour mechanisms and subsequent tumour development being mediated by a Ts-cell-

FIG. 4.—Tumour incidence after varying doses of Meth-A cells in BALB/c female mice (12 per group) which had received cyclophosphamide (100 mg/kg i.p.) 24 h before tumour challenge with the range of cell doses shown. The tumour incidence fell from 100% to 8% with graded doses of $10^6$ to $10^1$ cells. Sneaking through was not demonstrated and the resistance to tumour growth appeared to be less than in intact mice.

dependent mechanism akin to low-zone tolerance.

This work was supported in part by the Robin Stuart Trust of Sydney University and the National Health and Medical Research Council of Australia. The authors are grateful to the department of Audiovisual Services at Royal Prince Alfred Hospital and to Ms Lindsay Gatenby who typed the manuscript.

### REFERENCES

ALLISON, A. C. (1977) Immunological surveillance of tumours. *Cancer Immunol. Immunother.*, **2**, 151.

BURNET, F. M. (1970) *Immunological Surveillance.* London, New York, Toronto, Sydney. Oxford: Pergamon Press..

CANTOR, H. & BOYSE, E. A. (1977) Regulation of cellular and humoral immunity by T cell subclasses. *Cold Spring Harbor Symp. Quant. Biol.*, **41**, 23.

FARRAM, E., FESTENSTEIN, H. & GIORGI, L. DE (1978) The role of antibody in the inhibition of the growth of Meth-A tumour in syngeneic experiments *in vivo* and *in vitro. Clin. Exp. Immunol.*, **33**, 377.

HELLSTRÖM, K. E. & HELLSTRÖM, I. (1974) Lymphocyte-mediated cytotoxicity and blocking serum activity to tumour antigens. *Adv. Immunol.*, **18**, 209.

JULIUS, M. H., SIMPSON, E. & HERZENBERG, L. A. (1973) A rapid method for the isolation of functional thymus-derived murine lymphocytes. *Eur. J. Immunol.*, **3**, 645.

KLEIN, G. (1966) Recent trends in tumor immunology. *Isr. J. Med. Sci.*, **2**, 135.

KOLSCH, E. & MENGERSEN, R. (1976) Low numbers of tumor cells suppress the host immune system. *Adv. Exp. Med. Biol.*, **66**, 431.

MENGERSEN, R., SCHICK, R. & KOLSCH, E. (1975) Correlation of "Sneaking Through" of tumor cells with specific immunological impairment of the host. *Eur. J. Immunol.*, **5**, 532.

MILLER, J. F. A. P. & SPRENT, J. (1971) Cell to cell interaction in the immune response. VI. Contribution of thymus-derived cells and antibody-forming cell precursors to immunological memory. *J. Exp. Med.*, **134**, 66.

MITCHISON, N. A. (1971) The relative ability of T and B lymphocytes to see protein antigen. In *Cell Interactions and Receptor Antibodies in Immune Responses.* Eds Makela *et al.* London: Academic Press. p. 249.

MOLLER, G. & MOLLER, E. (1976) Foreword: The concept of immunological surveillance against neoplasia. *Transplant. Rev.*, **28**, 3.

NAOR, D. (1979) Suppressor cells: Permitters and promoters of malignancy. *Adv. Cancer Res.*, **29**, 45.

OLD, L. J., BOYSE, E. A., CLARKE, D. A. & CARSWELL, E. A. (1962) Antigenic properties of chemically induced tumors. *Ann. N.Y. Acad. Sci.*, **101**, 80.

PERRY, L. L. & GREENE, M. I. (1981) T cell subset interactions in the regulation of syngeneic tumor immunity. *Fed. Proc.*, **40**, 39.

ROLLINGHOFF, M., STARZINSKI-POWITZ, A., PFIZENMAIER, K. & WAGNER, H. (1977) Cyclophosphamide-sensitive T lymphocytes suppress the *in vivo* generation of antigen-specific cytotoxic T lymphocytes. *J. Exp. Med.*, **145**, 455.

SEIGAL, S. (1956) *Non-parametric Statistic for the Behavioural Sciences.* New York: McGraw-Hill.